# Sodium Binding Stabilizes the Outward-Open State of SERT by Limiting Bundle Domain Motions

**DOI:** 10.3390/cells11020255

**Published:** 2022-01-12

**Authors:** Dániel Szöllősi, Thomas Stockner

**Affiliations:** Institute of Pharmacology, Center for Physiology and Pharmacology, Medical University of Vienna, Waehringerstr. 13a, 1090 Vienna, Austria; daniel.szoelloesi@meduniwien.ac.at

**Keywords:** human serotonin transporter, sodium binding, bundle domain, molecular dynamics simulations, outward-open state, conformational stabilization

## Abstract

The human serotonin transporter (hSERT) removes the neurotransmitter serotonin from the synaptic cleft by reuptake into the presynaptic nerve terminal. A number of neurologic diseases are associated with dysfunction of the hSERT, and several medications for their treatment are hSERT blockers, including citalopram, fluoxetine, and paroxetine. The substrate transport is energized by the high concentration of external NaCl. We showed through molecular dynamics simulations that the binding of NaCl stabilized the hSERT in the substrate-binding competent conformation, which was characterized by an open access path to the substrate-binding site through the outer vestibule. Importantly, the binding of NaCl reduced the dynamics of the hSERT by decreasing the internal fluctuations of the bundle domain as well as the movement of the bundle domain relative to the scaffold domain. In contrast, the presence of only the bound chloride ion did not reduce the high domain mobility of the apo state.

## 1. Introduction

The physiological function of the human serotonin transporter (hSERT) is the reuptake of serotonin from the synaptic cleft into the presynaptic nerve terminal, thereby regulating the physiologically important concentration of the neurotransmitter serotonin. Several neurological diseases are associated with a dysfunction of the SERT, including depression, anxiety, and obsessive-compulsive disorders [1]. The SERT has a rich pharmacology [2] with several medications including antidepressants such as fluoxetine, citalopram, and tricyclic antidepressants increasing the concentration of serotonin in the synaptic cleft by preventing the reuptake of serotonin and consequently the process of vesicle storage replenishment.

Structures of hSERT have been solved in the presence of inhibitors in three conformations [3,4], which are interpreted as representing the outward-open, the outward-occluded, and the inward-open states. These structures reveal that hSERT transitions between these states through the movement of the mobile bundle domain (consisting of transmembrane helix (TMH) 1, 2, 6, and 7) relative to the scaffold domain (TMH3, 4, 8, and 9), which anchors the transporter in the cellular membrane. These structures show that for the hSERT the movement of the bundle domain first closes the access path to the substrate-binding site S1 from the extracellular site and subsequently opens a path for the substrate release to the intracellular side. In our paper, we refer to the outward-open state as “O-Open” and the outward-occluded state as “O-Occ”. The binding of the substrate to the central substrate-binding site S1 of the O-Open conformation initiates substrate transport that first leads to substrate occlusion and then to the conversion to the inward-open conformation, where the substrate and ions are released to the cytosol. The hSERT returns to the outward-facing state with either a bound potassium ion or a proton [5,6].

For the small amino acid transporter LeuT, it was directly shown through single-molecule Förster Resonance Energy Transfer (FRET) that LeuT continuously oscillates between the inward-facing state and the outward-facing state in the absence of sodium. The binding of sodium stabilizes the outward-facing state at low sodium concentrations (5 mM) [7]. For the hSERT, the stabilization of the outward facing (O-Open or O-Occ) states by sodium binding from the extracellular space was shown indirectly using N-FRET measurements that detected the distance between the N-terminus and the C-terminus at the intracellular site [8]. The distances are smaller in the presence of sodium than in its absence and are interpreted as representing the outward-facing and inward-facing states, respectively. Consistently, binding of the inhibitors cocaine or paroxetine stabilizes the hSERT in a conformation where the N-terminus and the C-terminus are in close proximity. Biochemical and electrophysiological measurements revealed that the binding of substrate or an inhibitor from the extracellular side requires the presence of sodium [9,10]. Moreover, accessibility studies showed that a cysteine in the intracellular vestibule becomes inaccessible for the methanethiosulphonate-ethylammonium (MTSEA) reagent after inhibitor binding [11]. These data provide clear evidence for an outward-facing state in the presence of bound sodium ions and the inhibitor, but it remains difficult to discriminate between the O-Open and the O-Occ conformations.

Here, we showed through molecular dynamics simulations that in the absence of bound ions, the O-Open state of the hSERT was very mobile while the sodium binding stabilized the O-Open conformation. The sodium ions bound to the sodium-binding sites (NA1 and NA2) were key players in the hSERT stabilization by fixing the distances between TMH1a and TMH1b and positioning TMH6a via direct interactions. The structural stabilization by sodium affected both the internal fluctuations of the bundle domain as well as the movement of the bundle domain relative to the scaffold domain. We started simulations from both the O-Open and the O-Occ structures to investigate the relative stability of both states and to study conformational changes induced by ion binding. 

## 2. Materials and Methods

### 2.1. System Creation and Molecular Dynamics Simulations

We assembled 5 independently created systems for each of 6 different states of hSERT, consisting of the O-Occ or the O-Open conformations, each in the apo state, in presence of Cl^−^ only or bound by one Cl^−^ and two Na^+^ ions. The O-Open crystal structure and the O-Occ cryo-EM structure (PDB ID: 5I71 [3]; PDBID 6DZV [4]) were used for the system building. Missing side chains were added with the homology modeling software package MODELLER 9.20 [12,13], while the ions were transferred from the O-Open structure to the O-Occ structure, which was devoid of ions. The best 5 of 100 created models were selected based on the Discrete Optimized Protein Energy (DOPE) score, which assessed the quality of protein models. All hSERT models were converted into a coarse-grained (CG) representation applying the MARTINI force field [14,15,16] and then embedded into a membrane containing POPC and cholesterol (mole ratio: 70:30). The simulation box was filled with water and 150 mM NaCl. All simulations were carried out using GROMACS version 2019.2 [17]. The CG systems were simulated for 1 μs to equilibrate the membrane and subsequently converted to an all-atom representation [18]. The original protein models were reinserted into the membrane to remove the small structural changes introduced during the CG simulations and/or by the conversion procedure to the all atom representation. The membed procedure [19] was used to relax spurious local atom overlaps. The protein, the ions, and the solvent were described using the amber99sb-ildn force field [20], POPC, and cholesterol by Slipid [21,22]. To prevent the rebinding of the removed Na^+^ or Cl^−^, the solvent contained only neutralizing Cl^−^ ions. The dimensions of the final system were 10.05 nm and 10.05 nm (*x*- and *y*-axes) in the membrane plane and 10.21 nm (*z*-axis) normal to the membrane.

All systems were energy-minimized and equilibrated in four steps of 2.5 ns each by slowly releasing the position restraints (1000, 100, 10, and 1 kJ/mol/nm) on the Cα atoms of the hSERT and on the bound ions, if present. Production runs were carried out for 500 ns. The temperature was maintained at 310 K using the v-rescale (τ = 0.5 ps) thermostat [23], while separately coupling the protein, the membrane, and the solvent. The pressure was maintained at 1 bar using the Parrinello–Rahman barostat [24] in a semi-isotropic manner applying a coupling constant of 20.1 ps. Long-range electrostatic interactions were described using the smooth particle mesh Ewald method [25] by applying a cutoff of 0.9 nm. The van der Waals interactions were described using the Lennard-Jones potentials and applying a cutoff of 0.9 nm. The long-range corrections for energy and pressure were applied. The coordinates of all atoms were recorded every 5 ps. The complete set of the MD simulation parameters of the production run can be found in the supporting information.

### 2.2. Data and Statistical Analysis

Figures and statistical analyses were generated by the Groningen MAchine for Chemical Simulation (GROMACS) package version 2019.2, R and python scripts using the MDAnalysis package, v0.19.2 [26] and ggplot v3.3.5 [27]. The vestibule size of SERT was analyzed with the HOLE program v2.2.005 [28] with default parameters. For the visualization, Visual Molecular Dynamics (VMD) version 1.9.3 [29] and PyMOL version 1.8.4 [30] were used. In order to ensure the samples independence of the statistical analysis of MD data, the samples were taken every 20 ns.

## 3. Results

The first step of the transport cycle of neurotransmitter uptake is the binding of the co-transported transport-energizing ions. These ions are typically present in orders of magnitude higher concentration than substrates or inhibitors. Hence, ion binding to an empty returning transporter is the dominant process. For the same reason, substrates or inhibitors interact with ion-loaded transporters; thus, affinities and any further transport steps are determined by this state. Functional and biochemical assays revealed that Na^+^ binding stabilizes the outward-facing state of the hSERT [7,8,11,31,32], but it remains unclear which of the structurally determined outward-facing conformations (O-Open or O-Occ) is dominant in the presence and absence of the co-transported ions. We addressed these questions using extensive equilibrium molecular dynamics simulations to explore conformations and dynamics with atomistic details, to investigate the contribution by the co-transported ions and to quantify the associated energies and the forces stabilizing the respective conformations.

### 3.1. Overall Transporter Dynamics and Mobility of the Bundle Domain

**Figure 1:**
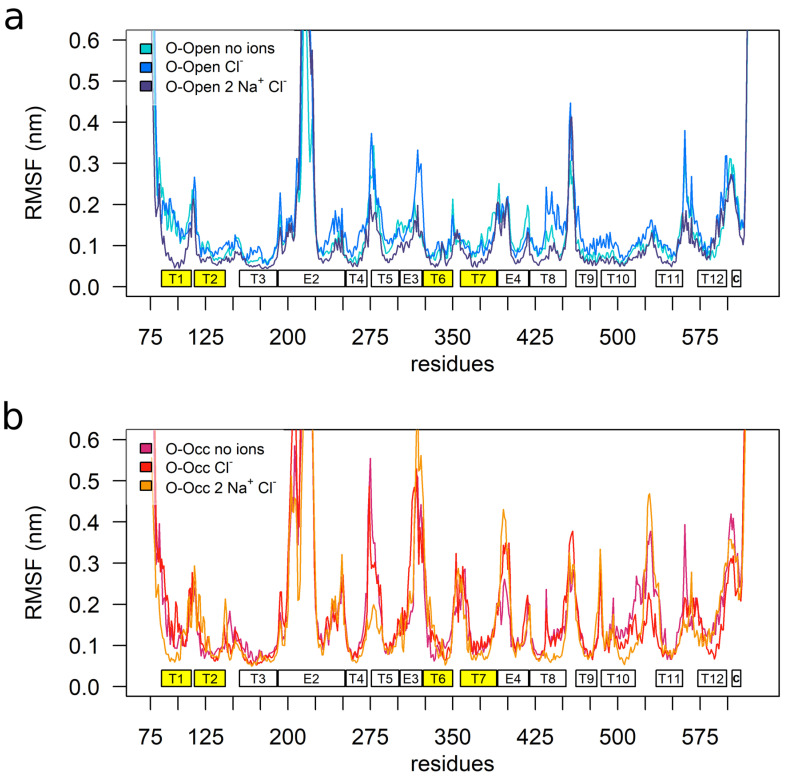
The dynamics of the human serotonin transporter (hSERT). Simulations starting from the O-Occ cryo-EM structure showed higher fluctuations as compared to the simulations starting from the O-Open structure. Root-mean-square fluctuation (RMSF) plots showed the mobility of Cα atoms of (**a**) the O-Open and (**b**) O-Occ conformations and their dependences on the bound ions, averaged over five independent parallel runs with a 1 ns temporal resolution.

To quantify the dynamics of the hSERT, we calculated the root-mean-square fluctuations (RMSFs) of the Cα atoms after fitting to the mean structure, in order to determine the fluctuation of each residue with respect to is mean position. The overall pattern of the hSERT mobility was aligned with the transporter topology (Figure 1), showing that the TMHs had lower mobility as compared to the intracellular and extracellular loops. While the patterns of the O-Open and the O-Occ conformations were similar, the extents of the observed motions were larger in the O-Occ conformation (Figure 1a,b). The middle part of extracellular loop 2 (EL2), specifically the region before the helix within EL2, showed exceptionally high fluctuations, consistent with earlier reports [33]. This larger mobility was expected, because the sequence and in the length of this section of the EL2 is not conserved between the 18 human paralogs of the solute carrier 6 (SLC6) family. Importantly, it had a sequence pattern typical for unstructured protein regions and was thus removed from the *Drosophila melanogaster* dopamine transporter before crystallization [34]. This loop region becomes glycosylated in the endoplasmic reticulum (ER) and the Golgi apparatus during maturation and trafficking to the plasma membrane.

Overall, the O-Occ conformation showed higher mobility than the O-Open conformation, indicating that the O-Open conformation might be the most stable state in the absence of the inhibitor or substrate. A comparison between the apo- and the Cl^−^-bound RMSF profiles of both the O-Open and the O-Occ conformations showed comparable profiles, indicating that the overall dynamics of hSERT was only minimally influenced by the presence of the Cl^−^ ion. Interestingly, TMH1b, TMH6a, and TMH7, which were in direct contact with the Cl^−^ ion, did not show significant local changes in dynamics upon Cl^−^ binding. In contrast, the presence of both Na^+^ ions and Cl^−^ reduced the overall mobility. This effect was stronger in the O-Open conformation, where the binding of Na^+^ significantly reduced the fluctuations. Particularly affected are the bundle domain (TMH1, TMH2, TMH6, and TMH7), and TMH8. This change in dynamics indicated that Na^+^ binding stabilized the conformation of the bundle domain in the O-Open conformation. Interestingly, in the O-Open conformation, the structural elements surrounding the entry path to the open outer vestibule (TMH1b, EL3, EL4, and EL5) showed particularly large dynamics, with TMH6a being an exception. The large dynamics of EL4a related most likely to its function, as this loop changed the conformation during the transport cycle. It is in line with experimental data reporting high deuterium-exchange rates [31,35] and showing high accessibility using the substituted-cysteine accessibility method (SCAM) [36].

### 3.2. The Bound Sodium Ions Stabilize TMH1b

**Figure 2:**
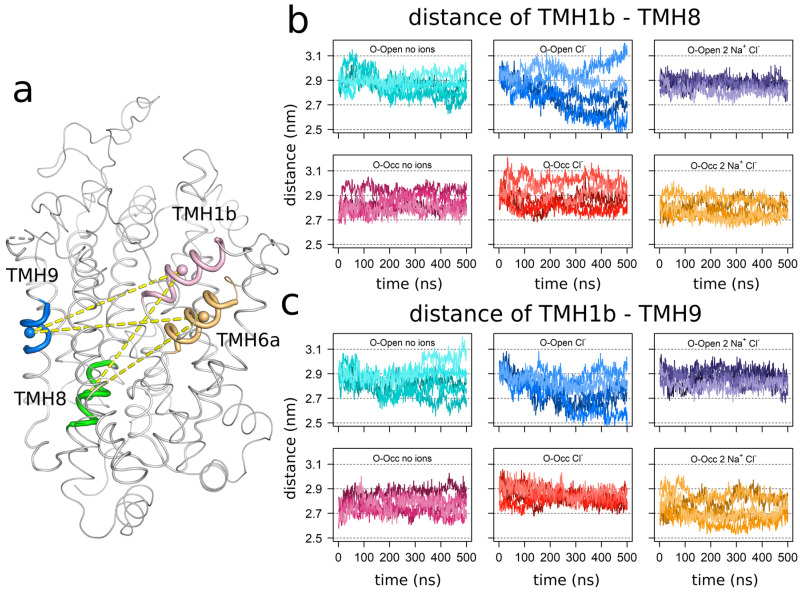
Bound ions stabilize transmembrane helix 1b (TMH1b). (**a**) Graphical legend highlighting the measured distances. The center of mass of TMH1b (residues 99–111), TMH6a (residues 328–338), TMH8 (residues 442–450), and TMH9 (residues 471–477) were used for measurements. These are highlighted as spheres. The time evolutions of the distances between TMH1b and TMH8 (**b**) and between TMH1b and TMH9 (**c**) are shown. All data were sampled with a 1 ns temporal resolution. The respective distances from TMH6a are shown in Supplementary Figure S1.

Next, we focused specifically on the dynamics of the bundle domain by measuring the distances of TMH1b (residues 99–108) (Figure 2) and TMH6a (residues 328–338) (Supplementary Figure S1) to the following: (i) the core region of TMH8 (residues 442–450) that was located at the bottom of the central substrate-binding site S1; (ii) the upper part of TMH9 (residues 471–477), located across the outer vestibule (Figure 2). The results for TMH6a were comparable to the results of TMH1b. The time evolutions (Figure 2b,c; Supplementary Figure S1) of the distances of TMH1b and TMH6a to TMH8 showed that these two helices can move closer to the substrate-binding site S1, if simulations starting from the O-Open conformation were carried out in the absence of the bound Na^+^. The distance distributions were generally broad, ranging from 2.6 to over 3.0 nm.

The dynamics was very different in the presence of bound Na^+^ ions (Levene’s test for the variance homogeneity of all O-Open simulations; *p* < 2.2 × 10^−16^). The O-Open conformation showed a strong restriction in the amplitude of the dynamic motions: the time evolution showed that five trajectories maintained the same distance to TMH8. In contrast, the simulations of the O-Occ state bound with the three ion (one Cl^−^ and two Na^+^ ions) were as dynamic as the respective Na^+^-free systems (Levene’s test for the variance homogeneity of the simulation of O-Occ with no ions vs. the simulations O-Occ with two Na^+^ and Cl^−^; *p* = 0.9736) and showed repeated movements towards a larger TMH8–TMH1b separation that resembled the O-Open conformation. The transition was not complete; however, TMH1b and TMH6a oscillated forth and back, indicating that much longer trajectories would be needed to obtain a complete convergence.

The distance across the vestibule towards TMH9 showed a similar pattern. The O-Open conformation becomes stable when all three ions are present (Levene’s test for the variance homogeneity of all O-Open simulations; *p* = 3.98 × 10^−8^). The observed distance reduction in the absence of bound Na^+^ ions was associated with vestibule closure. The O-Occ conformation was mobile and showed a particularly broad distribution in the presence of the three ions (Levene’s test for the variance homogeneity of all O-Occ simulations; *p* = 3.55 × 10^−6^), indicating a tendency to transition towards the O-Open conformation.

### 3.3. Correlation of TMH1b and TMH6a Movements

**Figure 3:**
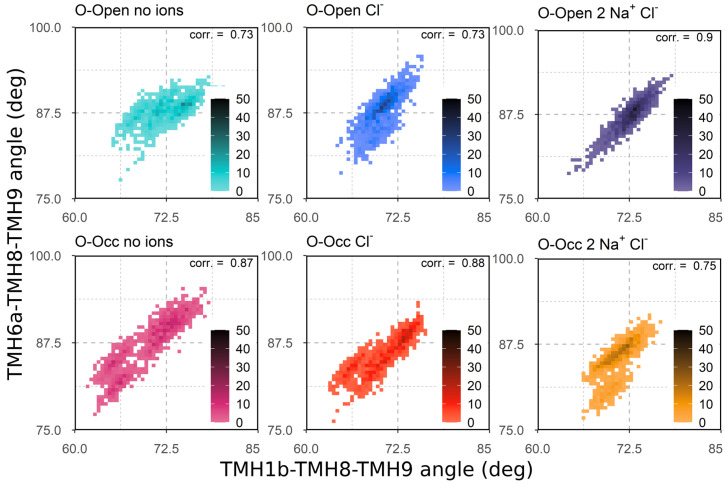
Ion dependences of the correlated motions of TMH1b and TMH6a. The angles between the center of mass of TMH1b–TMH8–TMH9 and TMH6a–TMH8–TMH9 are shown as two-dimensional (2D) histograms. The last 250 ns of 5 parallel simulations with a 1 ns temporal resolution were used. Angles are measured in degrees (deg). Pearson correlation coefficients (corr) are reported at the upper right corner of each plot.

To further characterize the motions of TMH1b and TMH6a, we measured the angles between TMH1b, TMH8, and TMH9 and the angles between TMH6a, TMH8, and TMH9 (Figure 3). These angles reported the degrees of the closure of the outer vestibule. If shown as a two-dimensional (2D) plot, the data also reveal the degree of correlation between the motions of the two helices (TMH1b and TMH6a), as data points accumulat along a diagonal if motions are correlated. Correlation coefficients are reported in Figure 3 using Pearson correlation applied to the last 250 ns of every simulation. The highest *p*-value obtained was 1.5 × 10^−11^. We found broader distributions in the absence of Na^+^ ions, which became particularly large when all three ions were absent. The results are consistent with the pattern determined for the distances (Figure 2).

In the presence of all three ions, these distributions were shifted to larger values for the TMH1b–TMH8–TMH9 angle (*x*-axis), but not for the TMH6a–TMH8–TMH9 angle (*y*-axis), indicating a conformational change of TMH1b only in response to the Na^+^ binding. The striking difference was the high degree of correlation in the motions of TMH1b and TMH6a of the O-Open conformation in the presence of Na^+^ ions. The high degree of correlation was visible by the much sharper distribution along the diagonal, also reflecting the structure stabilizing effect. The presence of the three ions did not induce the same degree of correlation in the O-Occ configuration, which was indicative of the structural instability of the occluded state, if in complex with the three ions.

### 3.4. Sodium Binding Reduces the Internal Dynamics of TMH1 and TMH6

**Figure 4:**
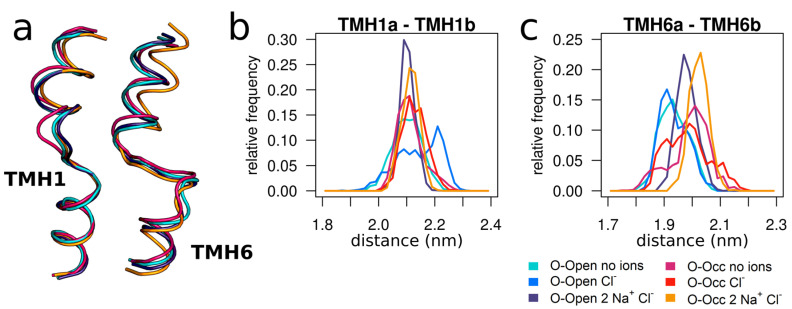
Na^+^ binding constrains the distances from TMH1a to TMH1b and from TMH6a to TMH6b. (**a**) Overlay of TMH1 and TMH6 (fitted to TMH1a and TMH6b) of representative structures. (**b**) Distance distributions of TMH1a (residues 86–95)–TMH1b (residues 99–111). (**c**) Distance distributions of TMH6a (residues 328–338)–TMH6b (residues 343–350). The last 250 ns of 5 parallel simulations with a 1 ns temporal resolution were used for the analysis.

The binding of the three ions reduced the overall and internal motions of the bundle domain in the O-Open state and the O-Occ state (Figure 1). These changes had a strong ordering effect on the relative distances between TMH1a (residues 86–95) and TMH1b and between TMH6a and TMH6b (residues 343–350) (Figure 4). The distribution of distances became narrower in the presence of Na^+^, visible by sharper and higher peaks in the distributions (the Levene’s test result for variance homogeneity is shown in Supplementary Table S1). Interestingly, while the centers of the distributions overlapped for TMH1 (Figure 4b), the binding of all three ions shifted the separation between TMH6a and TMH6b to a narrower distribution and to larger values as compared to the respective apo- and the Cl^—^only-bound states (tested by one-way ANOVA for all groups using the last 250 ns of simulations; *p* = 1.31 × 10^−5^).

### 3.5. Sodium Binding Decreases the Intradomain Mobility of the Bundle Domain of the O-Open Conformation

**Figure 5:**
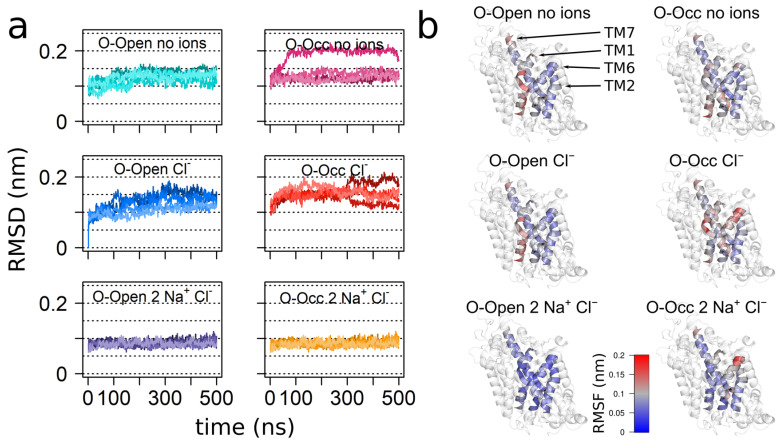
Internal motions of the bundle domain. The internal mobility of the bundle domain decreased, if in the O-Open state and in complex with one Cl^−^ and two Na^+^ ions: (**a**) root-mean-square deviation (RMSD) of the bundle domain after fitting the trajectories to the bundle domain using a 1 ns temporal resolution; (**b**) RMSF of the bundle domain (after fitting to the bundle domain), projected to the mean structure and color coded by the magnitude of fluctuations.

Next, we investigated how the binding of ions affects the internal conformation of the bundle domain. We used root-mean-square deviation (RMSD) analysis to measure the deviation of the bundle domain from its starting structure by fitting the trajectories to the respective starting conformation of the bundle domain before analyses. The Cl^−^-bound hSERT showed the strongest deviations in both the O-Open and the O-Occ states (Figure 5a). Deviations of the apo state were only minimally smaller. The binding of two Na^+^ and Cl^−^ ions stabilized the starting conformation of the bundle domain in the O-Open state, while the bundle domain of the O-Occ conformation deviated as much from the starting structure as the Cl^−^-only bound hSERT. Differences in the RMSD were statistically tested by one-way ANOVA using the last 250 ns of every simulation. The obtained *p*-value was less than 2 × 10^−16^. The pairwise comparison using Tukey post-hoc analysis is shown in Supplementary Figure S2.

Consistent with the RMSD data, the internal fluctuations measured by the RMSFs of the bundle domain were lower, if the hSERT was in the O-Open conformation and bound by one Cl^−^ and two Na^+^ ions (Figure 5b). The large amplitude of internal motions of the initial part of TMH6a, which was visible in the O-Occ conformation in the presence of Cl^−^ or if bound to all three ions, reflected the bundle domain internal rotation of TMH6a. This motion was associated with the movements of the O-Occ conformation towards the O-Open state. Similar changes were not visible for the O-Open state when in the same ion-binding state. In contrast to the O-Occ state, the O-Open state showed the largest mobility of TMH1a and TMH1b when the sodium ions were not present, while the internal fluctuations of the bundle domain were strongly reduced, once all three ions were present.

### 3.6. Analysis of the Global Motions of the Bundle Domain Shows That Ion Binding Restrains the Overall Bundle Domain Motions

**Figure 6:**
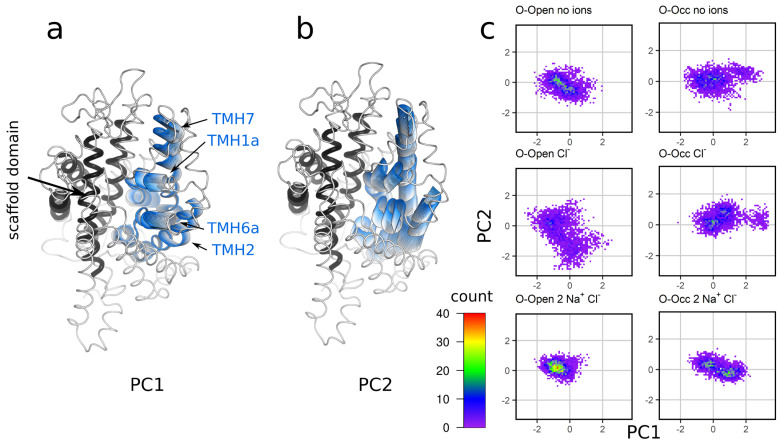
Principal component analysis indicates the reduced dynamics of the bundle domain. Visualization of the hSERT movements captured by principle component 1 (PC1) (**a**) and PC2 (**b**) shown as a series of intermediate conformations interpolated between the two most extreme conformations observed in the simulation of the O-Open Cl^−^-bound system. (**c**) Projection of the motions of the hSERT onto PC1 and PC2. The motions of the bundle domain (Cα atoms) were analyzed after fitting to the scaffold domain. The analysis included all trajectories with a time resolution of 1 ns. PC1 described 39% of the total variance, and PC2 described 20% of the total variance.

Next, we analyzed the overall dynamics of the bundle domain in response to ion binding using principal component analysis (PCA), which is a dimension reduction method that allows for the identification of the largest collective motions as principal components. The trajectories were fitted to the Cα positions of the scaffold domain, and the motions of the bundle domain were analyzed (Figure 6). The first principal component (PC1) was associated with motions that led to the opening/closing of the outer vestibule (Figure 6a), while PC2 was associated with the movements perpendicular to PC1 (Figure 6b). These PCs showed that the mobility of the bundle domain was largest when only Cl^−^ was bound, showing intermediate amplitudes for the motions of the apo state. The O-Open conformation of the Na^+^ and Cl^−^-bound hSERT showed a single strong peak, consistent with a single stable conformation. The two peaks present in the O-Occ state indicated that the hSERT was conformationally not stable in the O-Occ state, showing transitions to the O-Open conformation. Differences in the variance of all groups were tested by Levene’s test for the variance homogeneity and were significant with *p*-values of 3.7 × 10^−12^ for PC1 and 2.2 × 10^−16^ for PC2.

### 3.7. The Size of the Vestibule Indicates That Na^+^ Stabilizes an Open Access Path to the S1

**Figure 7:**
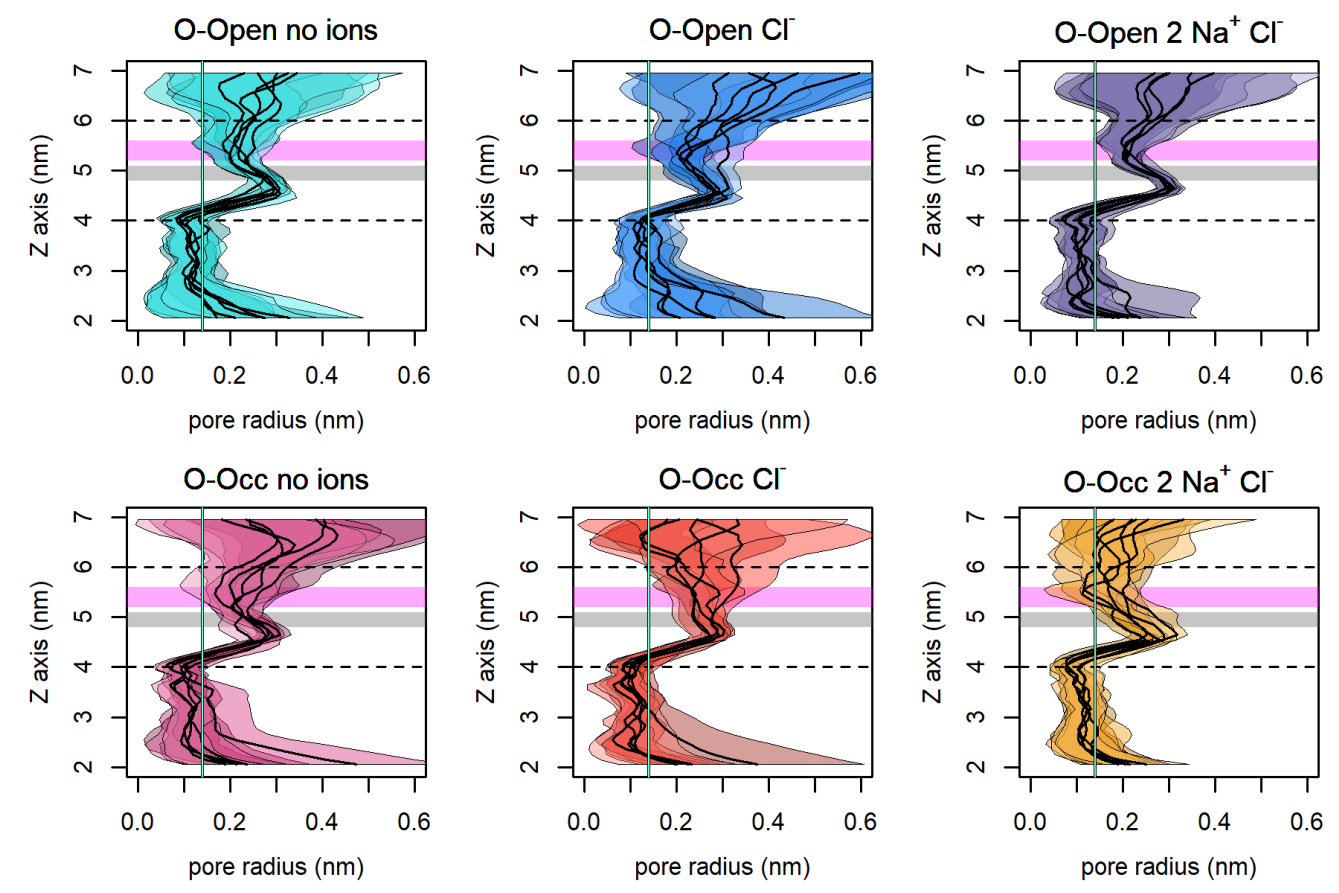
Mean pore radius ± standard deviation along the *z*-axis for every trajectory. The gray area indicates the extracellular hydrophobic lid, and the magenta area represents the extracellular salt bridge. The dashed lines show the bottom of the S1 binding pocket (at 4 nm) and the outer end of the extracellular vestibule (at 6 nm). The aquamarine vertical line indicates the radius of a water molecule (0.14 nm) for a comparison. The vestibule was considered closed if smaller than the size of a water molecule. Trajectories were analyzed every 1 ns.

The stabilization of an open outer vestibule was important for the binding of the substrate and co-transported ions to the S1. We measured the mean radius and the fluctuation of the vestibule size along the *z*-axis to estimate the accessibility of the S1 using the HOLE program (Figure 7), which allowed for the quantification of the size of pores in protein structures using a radial measure. The dashed horizontal lines indicate the bottom of the S1 (at 4 nm) and the entry to the outer vestibule (at 6 nm). The aquamarine vertical line indicates the radius of a water molecule (0.14 nm), which serves to indicate a complete closure of the passage. Consistent with previous measures, Figure 7 shows that the O-Open hSERT in complex with Na^+^ and Cl^−^ maintained the most widely open outer vestibule and showed the lowest fluctuations of the outer vestibule. The conformational stability of the open conformation was highlighted by the low amplitude of motions at the hydrophobic gate and the extracellular salt bridge (R104-E493), which are highlighted as gray and magenta areas, respectively.

## 4. Discussion

Most small molecules including vitamins, ions, sugars, and amino acids need carriers to cross cell membranes. These transporters are often secondary active transporters in eukaryotic cells. Secondary active transporters share the mechanistic principle of substrate transport, which is the alternating access mechanism [37]. The inverted repeat [38] appears to be a common structural element in SLC transporters that facilitates the alternation of the accessibility to the substrate-binding site located in the center of the transmembrane domain. In SLC6 transporters, the access to the substrate-binding site is regulated by the bundle domain. 

Human monoamine transporters oscillate between the outward-facing and the inward-facing conformations in the absence of Na^+^, while the outward-facing conformation is stabilized by Na^+^ binding [5,39]. Chloride is essential for transport, but it remains disputed if Cl^−^ contributes to the driving force [5,40,41]. The directionality of the transport process is controlled by the transmembrane concentration gradient of the co-transported Na^+^ ion(s). The hSERT uses a symport of Na^+^ to energize the cellular uptake of serotonin. It remains unclear how Na^+^ binding stabilizes the outward-facing conformation and if the O-Open conformation is preferred over the O-Occ conformation.

In this study, we investigated the impact of the co-transported Na^+^ and Cl^−^ on the conformation of the hSERT. The stabilization of the O-Open conformation was counter-intuitive from a naïve perspective, because it stabilized a conformation that was geometrically most distant from the inward-open state, while sodium primed SLC6 transporters for going from the O-Open to the inward-open conformations in the presence of a substrate. We found that the O-Occ conformation was conformationally unstable in the apo state, in the presence of only Cl^−^, but also when in complex with one Cl^−^ and two Na^+^ ions. In contrast, we found that Cl^−^ and Na^+^ binding led to the stabilization of the O-Open state and to a reduction of intradomain as well as the interdomain mobility of the bundle domain.

Several lines of evidence have reported that Na^+^ binding stabilizes an outward-facing conformation (O-Open or O-Occ). The presence of Na^+^ is required for substrate and inhibitor binding, as shown by biochemical and electrophysiological measurements of the hSERT [9,10]. The distance measurement between the N-terminus and the C-terminus using N-FRET revealed a tendency towards shorter distances in the presence of Na^+^, which were similar to the short distances observed after inhibitor (cocaine and paroxetine) binding [8]. In the case of LeuT, FRET experiments showed a decreased transition frequency with a preference for the outward-facing state upon increasing the sodium concentration [7]. Similarly, the electron paramagnetic resonance (EPR) measurements of the ion- and substrate-free LeuT showed a free transitioning between the outward-facing and inward-facing conformations while the binding of sodium stabilizes the outward-facing conformation [39]. Hydrogen–deuterium exchange mass spectrometry experiments on the hSERT [35] and the *Drosophila melanogaster* dopamine transporter [31] indicated the increased stability of TM1a, TM6a, and IL4 in the presence of Na^+^, while higher deuterium exchange was measured at TM1b, supporting the O-Open stabilization effect of Na^+^.

Molecular dynamics simulations [42] of Mhp1 (a LeuT-fold, Na^+^-coupled secondary transporter) concluded that sodium stabilizes the O-Open state by restraining the relative motion between the scaffold and the bundle domain. Consistent with our data, computational studies of LeuT starting from the Na^+^-bound O-Occ state showed a spontaneous conversion to the O-Open state [43,44,45].

We showed that Na^+^ binding to the hSERT selected the O-Open conformation, stabilized it and reduced fluctuation. Stabilizing the hSERT in the O-Open conformation by extracellular Na^+^ supported the binding and transport of substrate by increasing the number of transporters in a substrate-binding competent O-Open conformation. It also stabilized the outer vestibule in its most open conformation, which is important for the access of the substrate 5HT to the S1, thereby increasing the on-rate for substrates and its affinity. The binding of substrate to the S1 leads to its occlusion by the movement of the bundle domain, which strongly reduces the off-rate by closing the escape path to the extracellular side of the membrane. In addition, Na^+^ increased the affinity of substrates for hSERT by orders of magnitude [46], as Na^+^ bound to the NA1 sodium-binding site was in very close proximity to the positively charged amino group of 5HT, stabilizing the bound substrate by strong electrostatic interactions. We inferred from these considerations and our data that sodium plays two roles in the first part of the transport cycle as following: (i) it stabilizes the substrate-binding competent O-Open conformation that allows for efficient substrate access; and (ii) it strongly increases the affinity of the substrate 5HT to the hSERT. The combination of these factors guarantees that the transport complex consisting of the hSERT, Na^+^, Cl^−^, and substrate assembles before the hSERT converts to the inward-open state, preventing futile cycling. The release of the substrate to the cytosol is assured, as long as the concentrations of the substrate and sodium in the cytosol are below their affinities to the cytosolic side of the hSERT.

## Data Availability

All data are deposited in https://zenodo.org, doi:10.5281/zenodo.5668265.

## Supplementary Materials

The following are available online at https://www.mdpi.com/article/10.3390/cells11020255/s1, Figure S1: The Na^+^ ions stabilize TMH6a; The Molecular Dynamics Parameters (MDP) file of the production simulation; Figure S2: One-way ANOVA and Tukey post-hoc analysis of the bundle domain RMSD; Table S1: Levene’s test for variance homogeneity of TM1a–TM1b and TM6a–TM6b distances.
