# Supplementary material for: Sodium Binding Stabilizes the Outward-Open State of SERT by Limiting Bundle Domain Motions
Source: Cells. 2022 Jan 12;11(2):255. doi: 10.3390/cells11020255 (PMC8773566; doi:10.3390/cells11020255)
Supplement: Supplementary file 1 [file cells-11-00255-s001.zip › cells-1482423-supplementary.pdf]

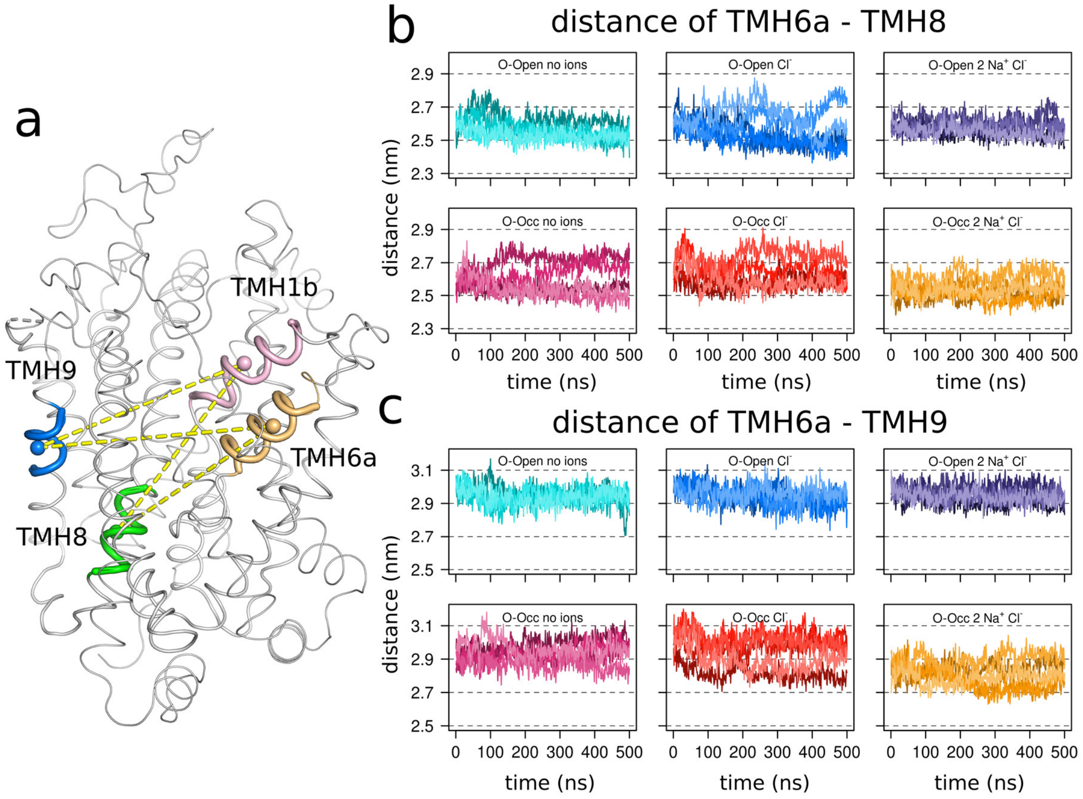

Figure S1. The Na^+ ions stabilize TMH6a. (**a**) Graphical legend highlighting measured distances. The segments of TMH6a (residue 328–338), TMH8 (residue 442–450) and TMH9 (residue 471 to 477), which are used for the distance measurements, are highlighted and their respective center of mass is visualized by a sphere. Panel (**b**) and (**c**) show the time evolution of distances (**b**) between TMH6a and TMH8 and (**c**) between TMH1b and TMH9. All data are sampled with a 1 ns temporal resolution.

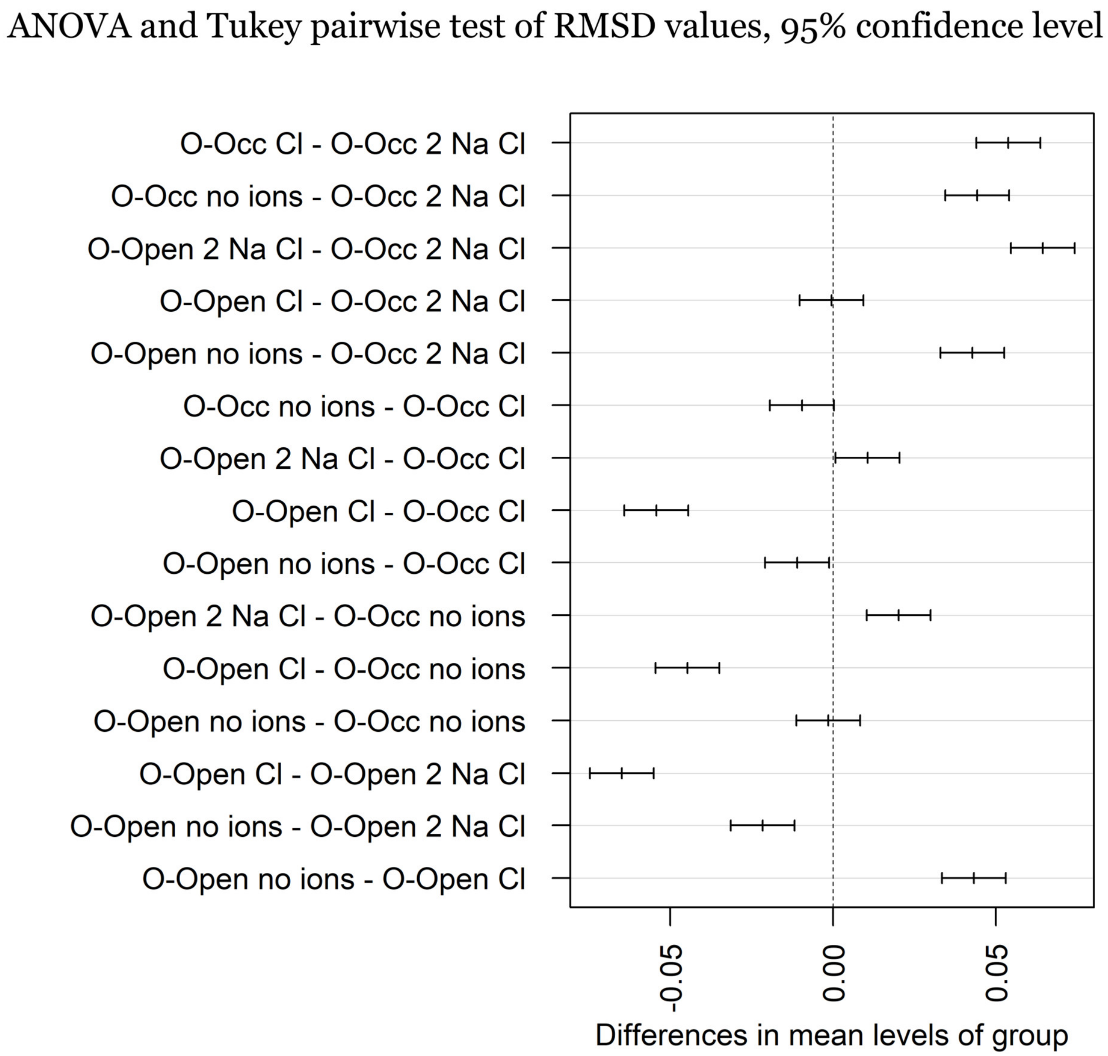

Figure S2. One-way ANOVA and Tukey post-hoc analysis of the bundle domain RMSD. The analysis used the last 250 ns of each simulation. The obtained p value of the ANOVA is less than 2×10^{-16}.

Table S1. Levene's test for the variance homogeneity of TM1a – TM1b and TM6a – TM6b distances.

	TMH1a – TMH1b	TMH6a – TMH6b
	p-value	*p*-value
comparing all O-Open	0.002308	6.08×10^{-9}
comparing all O-Occ	0.0008307	4.42×10^{-8}

Production MDP file

This parameter file was used for controlling GROMACS during the production simulations

```
; VARIOUS PREPROCESSING OPTIONS
; Preprocessor information: use cpp syntax.
; e.g.: -I/home/joe/doe -I/home/mary/roe
;include                  =
; e.g.: -DPOSRES -DFLEXIBLE (note these variable names are case sensitive)
;define                   =

; RUN CONTROL PARAMETERS
integrator               = md
; Start time and timestep in ps
tinit                    = 0
dt                       = 0.002
nsteps                   = 250000000 ; 0.5 us
; For exact run continuation or redoing part of a run
init_step                = 0
; Part index is updated automatically on checkpointing (keeps files separate)
simulation_part          = 1
; mode for center of mass motion removal
comm-mode                = linear
; number of steps for center of mass motion removal
nstcomm                  = 100
; group(s) for center of mass motion removal
comm-grps                = system

; LANGEVIN DYNAMICS OPTIONS
; Friction coefficient (amu/ps) and random seed
bd-fric                  = 0
ld-seed                  = 1993

; ENERGY MINIMIZATION OPTIONS
; Force tolerance and initial step-size
emtol                    = 1000
emstep                   = 0.0001
; Max number of iterations in relax-shells
niter                    = 20
; Step size (ps^2) for minimization of flexible constraints
fcstep                   = 0.001
; Frequency of steepest descents steps when doing CG
nstcgsteep               = 50
nbfgscorr                = 100

; TEST PARTICLE INSERTION OPTIONS
rtpi                     = 0.05

; OUTPUT CONTROL OPTIONS
; Output frequency for coords (x), velocities (v) and forces (f)
nstxout                  = 2500 ; 5 ps
nstvout                  = 2500
```

```
nstfout                  = 0
; Output frequency for energies to log file and energy file
nstlog                   = 1000
nstcalcenergy            = 100
nstenergy                = 1000
; Output frequency and precision for .xtc file
nstxout-compressed       = 0
compressed-x-precision   = 1000
; This selects the subset of atoms for the compressed
; trajectory file. You can select multiple groups. By
; default, all atoms will be written.
compressed-x-grps        =
; Selection of energy groups
energygrps               =

; NEIGHBORSEARCHING PARAMETERS
; cut-off scheme (Verlet: particle based cut-offs, group: using charge groups)
cutoff-scheme            = Verlet
; nblist update frequency
nstlist                  = 50
; ns algorithm (simple or grid)
ns-type                  = Grid
; Periodic boundary conditions: xyz, no, xy
pbc                      = xyz
periodic_molecules       = no
; Allowed energy error due to the Verlet buffer in kJ/mol/ps per atom,
; a value of -1 means: use rlist
verlet-buffer-tolerance  = 0.005
; nblist cut-off
rlist                    = 0.9
; long-range cut-off for switched potentials
rlistlong                = -1
nstcalclr                = -1

; OPTIONS FOR ELECTROSTATICS AND VDW
; Method for doing electrostatics
coulombtype              = PME
coulomb-modifier         = Potential-shift-Verlet
rcoulomb-switch          =
rcoulomb                 = 0.9
; Relative dielectric constant for the medium and the reaction field
epsilon_r                = 1.0
epsilon_rf               = 1
; Method for doing Van der Waals
vdw-type                 = Cut-off
vdw-modifier             = Potential-shift-Verlet
; cut-off lengths
rvdw-switch              =
rvdw                     = 0.9
; Apply long range dispersion corrections for Energy and Pressure
DispCorr                 = EnerPres
; Extension of the potential lookup tables beyond the cut-off
table-extension          = 1
```

```
; Separate tables between energy group pairs
energygrp-table          =
; Spacing for the PME/PPPM FFT grid
fourierspacing           = 0.12
; FFT grid size, when a value is 0 fourierspacing will be used
fourier_nx               = 0
fourier_ny               = 0
fourier_nz               = 0
; EWALD/PME/PPPM parameters
pme_order                = 4
ewald_rtol               = 1e-05
ewald-rtol-lj            = 0.001
lj-pme-comb-rule         = Geometric
ewald_geometry           = 3d
epsilon_surface          = 0

; IMPLICIT SOLVENT ALGORITHM
implicit_solvent         = No

; GENERALIZED BORN ELECTROSTATICS
; Algorithm for calculating Born radii
gb-algorithm             = Still
; Frequency of calculating the Born radii inside rlist
nstgbradii               = 1
; Cutoff for Born radii calculation; the contribution from atoms
; between rlist and rgbradii is updated every nstlist steps
rgbradii                 = 1
; Dielectric coefficient of the implicit solvent
gb-epsilon-solvent       = 80
; Salt concentration in M for Generalized Born models
gb-saltconc              = 0
; Scaling factors used in the OBC GB model. Default values are OBC(II)
gb-obc-alpha             = 1
gb-obc-beta              = 0.8
gb-obc-gamma             = 4.85
gb-dielectric-offset     = 0.009
sa-algorithm             = Ace-approximation
; Surface tension (kJ/mol/nm^2) for the SA (nonpolar surface) part of GBSA
; The value -1 will set default value for Still/HCT/OBC GB-models.
sa-surface-tension       = -1

; OPTIONS FOR WEAK COUPLING ALGORITHMS
; Temperature coupling
tcoupl                   = v-rescale
nsttcouple               = -1
nh-chain-length          = 10
print-nose-hoover-chain-variables = no
; Groups to couple separately
tc-grps                  = ProtLigIon membrane Water_and_ions
; Time constant (ps) and reference temperature (K)
tau-t                    = 0.5   0.5   0.5
ref-t                    = 310   310   310
; pressure coupling
```

```
Pcoupl                   = Parrinello-Rahman
Pcoupltype               = Semiisotropic
nstpcouple               = -1
; Time constant (ps), compressibility (1/bar) and reference P (bar)
tau-p                    = 20.1
compressibility          = 4.5e-05   4.5e-05
ref-p                    = 1.0       1.0
; Scaling of reference coordinates, No, All or COM
refcoord_scaling         = All

; OPTIONS FOR QMMM calculations
QMMM                     = no
; Groups treated Quantum Mechanically
QMMM-grps                =
; QM method
QMmethod                 =
; QMMM scheme
QMMMscheme               = normal
; QM basisset
QMbasis                  =
; QM charge
QMcharge                 =
; QM multiplicity
QMmult                   =
; Surface Hopping
SH                       =
; CAS space options
CASorbitals              =
CASelectrons             =
SAon                     =
SAoff                    =
SAsteps                  =
; Scale factor for MM charges
MMChargeScaleFactor      = 1
; Optimization of QM subsystem
bOPT                     =
bTS                      =

; SIMULATED ANNEALING
; Type of annealing for each temperature group (no/single/periodic)
annealing                = no
; Number of time points to use for specifying annealing in each group
annealing-npoints        =
; List of times at the annealing points for each group
annealing-time           =
; Temp. at each annealing point, for each group.
annealing-temp           =

; GENERATE VELOCITIES FOR STARTUP RUN
gen-vel                  = no
gen-temp                 = 310.0
gen-seed                 = -1
```

```
; OPTIONS FOR BONDS
constraints              = h-bonds
; Type of constraint algorithm
constraint-algorithm     = lincs
; Do not constrain the start configuration
continuation             = no
; Use successive overrelaxation to reduce the number of shake iterations
Shake-SOR                = yes
; Relative tolerance of shake
shake-tol                = 0.0001
; Highest order in the expansion of the constraint coupling matrix
lincs-order              = 4
; Number of iterations in the final step of LINCS. 1 is fine for
; normal simulations, but use 2 to conserve energy in NVE runs.
; For energy minimization with constraints it should be 4 to 8.
lincs-iter               = 2
; Lincs will write a warning to the stderr if in one step a bond
; rotates over more degrees than
lincs-warnangle          = 30
; Convert harmonic bonds to morse potentials
morse                    = no

; ENERGY GROUP EXCLUSIONS
; Pairs of energy groups for which all non-bonded interactions are excluded
energygrp-excl           =

; WALLS
; Number of walls, type, atom types, densities and box-z scale factor for Ewald
nwall                    = 0
wall_type                = 9-3
wall_r_linpot            = -1
wall-atomtype            =
wall-density             =
wall_ewald_zfac          = 3

; COM PULLING
; Pull type: no, umbrella, constraint or constant-force
pull                     = no

; ENFORCED ROTATION
; Enforced rotation: No or Yes
rotation                 = no

; Group to display and/or manipulate in interactive MD session
IMD-group                =

; NMR refinement stuff
; Distance restraints type: No, Simple or Ensemble
disre                    = No
; Force weighting of pairs in one distance restraint: Conservative or Equal
disre-weighting          = Conservative
; Use sqrt of the time averaged times the instantaneous violation
disre-mixed              = no
```

```
disre-fc                 = 100
disre-tau                = 0
; Output frequency for pair distances to energy file
nstdisreout              = 5000
; Orientation restraints: No or Yes
orire                    = no
; Orientation restraints force constant and tau for time averaging
orire-fc                 = 0
orire-tau                = 0
orire-fitgrp             =
; Output frequency for trace(SD) and S to energy file
nstorireout              = 100

; Free energy variables
free-energy              = no
couple-moltype           =
couple-lambda0           = vdw-q
couple-lambda1           = vdw-q
couple-intramol          = no
init-lambda              = 0
init-lambda-state        = -1
delta-lambda             = 0
nstdhdl                  = 50
fep-lambdas              =
mass-lambdas             =
coul-lambdas             =
vdw-lambdas              =
bonded-lambdas           =
restraint-lambdas        =
temperature-lambdas      =
calc-lambda-neighbors    = 1
init-lambda-weights      =
dhdl-print-energy        = no
sc-alpha                 = 0
sc-power                 = 1
sc-r-power               = 6
sc-sigma                 = 0.3
sc-coul                  = no
separate-dhdl-file       = yes
dhdl-derivatives         = yes
dh_hist_size             = 0
dh_hist_spacing          = 0.1

; Non-equilibrium MD stuff
acc-grps                 =
accelerate               =
freezegrps               =
freezedim                =
cos-acceleration         = 0
deform                   =

; simulated tempering variables
simulated-tempering      = no
```

```
simulated-tempering-scaling = geometric
sim-temp-low             = 300
sim-temp-high            = 300

; Electric fields
; Format is number of terms (int) and for all terms an amplitude (real)
; and a phase angle (real)
E-x                      =
; Time dependent (pulsed) electric field. Format is omega, time for pulse
; peak, and sigma (width) for pulse. Sigma = 0 removes pulse, leaving
; the field to be a cosine function.
E-xt                     =
E-y                      =
E-yt                     =
E-z                      =
E-zt                     =

; Ion/water position swapping for computational electrophysiology setups
; Swap positions along direction: no, X, Y, Z
swapcoords               = no

; AdResS parameters
adress                   = no

; User defined thingies
user1-grps               =
user2-grps               =
userint1                 = 0
userint2                 = 0
userint3                 = 0
userint4                 = 0
userreal1                = 0
userreal2                = 0
userreal3                = 0
userreal4                = 0
```
